# Role of corneal epithelial thickness during myopic regression in femtosecond laser-assisted in situ keratomileusis and transepithelial photorefractive keratectomy

**DOI:** 10.1186/s12886-022-02727-x

**Published:** 2022-12-08

**Authors:** Hua Li, Qichao Han, Jiafan Zhang, Ting Shao, Huifeng Wang, Keli Long

**Affiliations:** 1 Eye Institute of Shandong First Medical University, Qingdao Eye Hospital of Shandong First Medical University, State Key Laboratory Cultivation Base, Shandong Provincial Key Laboratory of Ophthalmology, School of Ophthalmology, Shandong First Medical University, 266071 Qingdao, Shandong Province China; 2 Department of Ophthalmology, Zaozhuang Municipal Hospital, 277100 Zaozhuang, Shandong Province China

**Keywords:** Corneal epithelial thickness, FS-LASIK, TPRK, Myopic regression

## Abstract

**Background:**

The study aimed to investigate the relationship between changes in corneal epithelial thickness and the outcome of myopic regression after femtosecond laser-assisted in situ keratomileusis (FS-LASIK) and transepithelial photorefractive keratectomy (TPRK).

**Methods:**

This study included 45 eyes of 25 patients undergoing FS-LASIK and 44 eyes of 24 patients undergoing TPRK. Myopic regression occurred in these patients postoperatively from 8 to 21 months. The corneal epithelial thickness was measured using a spectral-domain optical coherence tomography at the onset of regression, 3 months after treatment, and 3 months after drug withdrawal.

**Results:**

Compared with that of preoperation, corneal epithelial thickness increased when regression occurred in both groups (all *P* < 0.05). The thickness of central corneal epithelium in FS-LASIK and TPRK groups reached 65.02 ± 4.12 µm and 61.63 ± 2.91 µm, respectively. The corneal epithelial thickness decreased when myopic regression subsided after 3 months of steroid treatment compared to the onset (*P* < 0.05). With a decrease in corneal epithelial thickness, the curvature of the anterior corneal surface, central corneal thickness, and refractive power all decreased (all *P* < 0.05). The corneal epithelial thickness and refractive error remained relatively stable after 3 months of treatment withdrawal (*P* > 0.05).

**Conclusion:**

The corneal epithelial thickness determined the outcome of myopic regression similarly in FS-LASIK and TPRK. When the corneal epithelium thickened, regression occurred. After steroid treatment, epithelial thickness decreased whereas regression subsided.

## Background

Uncorrected myopia is one of the main causes of visual impairment worldwide. Since the 1990s, corneal refractive surgery has become a safe and effective method for correcting myopia, which has a long-term and stable outcome. However, refractive regression after surgery often occurs in patients with high myopia [1–4]. Previous studies have reported that the rate of regression in high myopic eyes is approximately 30% [5]. Refractive regression affects the predictability, effectiveness, and long-term stability of surgery and is one of the main causes for patients’ dissatisfaction with surgery. In addition to the degree of preoperative refractive error, the occurrence of postoperative regression is related to many factors, such as age, dry eye, the corneal curvature gradient, the thickness of corneal flap, residual stromal bed, and diameter of ablating optical zone [6–8]. Corneal epithelial hyperplasia, the protruding of corneal posterior surface, corneal stromal reaction, wound healing response, and decreased biomechanical stability may cause refractive regression after surgery, but the exact mechanism of regression remains unclear [9].

Recently, the corneal epithelium has been found to thicken in the laser ablating zone after laser-assisted in situ keratomileusis (LASIK), photorefractive keratectomy (PRK), and small-incision lenticule extraction (SMILE), resulting in refractive regression [10, 11]. The degree of epithelial thickening is significantly correlated with the degree of preoperatively myopic correction [12, 13]. The higher the degree of myopic correction, the greater the corneal curvature gradient after surgery and higher the possibility of epithelial thickening. Previous studies have reported that epithelial thickness after LASIK is directly related to the outcome of refractive status [14, 15]. Topical steroidal agents reducing corneal epithelial thickness were used to reduce refractive regression [16]. It is still unknown whether the change in epithelium thickness following steroid treatment in PRK patients with refractive regression is related to the improvement of refractive state and whether it differs from LASIK model. Furthermore, it is unclear whether refractive regression will occur and whether the epithelium will thicken again after the discontinuation of steroid treatment.

In this prospective study, we investigated the changes of epithelial thickness and refractive status after steroidal treatment in patients with refractive regression after femtosecond LASIK (FS-LASIK) and transepithelial PRK (TPRK). We also examined the changes in the two variables (FS-LASIK and TPRK) after the discontinuation of steroidal treatment.

### Subjects and methods

#### Study design and patients

We conducted a prospective interventional study. Patients with refractive regression during the follow-up after surgery in Qingdao Eye Hospital from October 2019 to January 2021 were enrolled in the study, including 25 patients (45 eyes) undergoing FS-LASIK and 24 patients (44 eyes) undergoing TPRK. The initial surgery occurred from June 2018 to December 2019. This study followed the tenets of the Declaration of Helsinki and was approved by the ethics committee of Qingdao Eye Hospital (Code Number: 2021-02). Informed consent was signed by all patients.

Refractive regression was defined as a myopic shift of 0.5 diopters (D: spherical equivalent) or greater at >= 3 months after surgery. An uncorrected distance visual acuity (UDVA) in enrolled patients should have reached 20/20 or more within 3 months after the initial surgery. Exclusion criteria were the following: (1) patients with other ocular diseases, such as glaucoma and dry eye; (2) active inflammation of ocular surface and anterior segment; (3) presence of systemic diseases; (4) under-correction in the initial surgery: (5) myopia caused by lens changes; and (6) iatrogenic corneal ectasia.

### Ocular examination

Before surgery, all patients underwent standard ophthalmic examinations, including UDVA, corrected distance visual acuity (CDVA), cycloplegic and manifest refraction, intraocular pressure, axis length, corneal curvature and thickness, corneal biomechanics using Corvis ST, corneal topography using Pentacam HR (Oculus, Wetzlar, Germany), and corneal epithelial thickness using anterior segment optical coherence tomography (Optovue Inc., Fremont, USA). Each epithelial thickness map covering a 7-mm diameter area was divided into 17 sectors, including a central 2-mm zone and eight octants equally distributed within paracentral (2–5 mm), midperipheral (5–7 mm), and annular zones. The corneal curvature was measured by Pentacam HR to evaluate the changes of corneal refractive power during the follow-up. Each examination was performed by the same experienced and qualified optometrist. The ocular measurements were performed preoperatively, at the beginning of drug treatment, at the end of treatment, and at 3 months after drug withdrawal.

### Surgical procedures and drug treatment

All surgeries were performed in the Amaris 750 S excimer laser platform with smart pulse technology (Schwind Eye-Tech-Solutions, Germany). The ablation zone was set to 5.9–6.3 mm and a transition zone of 1.5–2.0 mm. In order to prevent the formation of haze in TPRK group, it was recommended to take vitamins c orally and wear ultraviolet protective glasses for a few months after surgery. Patients with regression due to the formation of haze were not enrolled in the study. The enrolled patients were prescribed 0.1% flumirone eye drops (Santen, Tokyo, Japan) four times a day for the first month when refractive regression occurred. Following this, it was reduced to twice a day in the second month and once a day in the third month. The whole treatment lasted for 3 months. When the intraocular pressure increased by more than 5 mm Hg after steroid treatment in few patients, 2% carteolol hydrochloride eye drops were added.

#### Statistical analysis

All data were statistically analyzed using IBM SPSS version 26.0 (IBM Inc., New York, USA). The normality of the data was verified with Kolmogorov–Smirnov test. The non-normally distributed data were analyzed with Mann–Whitney test. Continuous variables were expressed in mean and standard deviation. Categorical variables were expressed as percentages. Independent sample *t* test was used for comparison between the two groups. Paired *t* test was used for intra-group analysis at different time points. Chi-square or Fischer-exact test was used to compare categorical variables. A *P* value of less than 0.05 was considered statistically significant.

## Results

This study included 25 patients (45 eyes, 7 males and 18 females, during the same period a total of 1591 eyes underwent FS-LASIK and the regression rate was 2.83%) with refractive regression in FS-LASIK group and an average age of 29.48 ± 4.90 years (21–41 years). There were 24 patients (44 eyes, 10 males and 14 females, a total of 1758 eyes underwent T-PRK and the regression rate was 2.50%) in TPRK group, with an average age of 26.67 ± 6.42 years (18–39 years). The preoperative spherical equivalent in FS-LASIK and TPRK groups were - 7.56 ± 1.01 D and - 7.20 ± 1.01 D, respectively. There were significant differences in the cylinderical diopter and corneal thicknesses between the two groups before surgery (*P* < 0.05) and no significant differences in other variables (*P* > 0.05). The postoperative onset time of myopic regression was 11.16 ± 3.18 months (8–21 months) in FS-LASIK group and 11.83 ± 2.70 months (8–19 months) in TPRK group (*P* > 0.05; Table 1).

**Table 1:** Comparisons of ocular parameters between FS-LASIK and TPRK groups before surgery (Mean ± SD)

Parameters	FS-LASIK (*n* = 45 eyes)	TPRK (*n* = 44 eyes)	*P*
Age (years)	29.48 ± 4.90	26.67 ± 6.42	0.090
UDVA (logMAR)	1.29 ± 0.26	1.31 ± 0.31	0.693
CDVA (logMAR)	0.01 ± 0.03	0.01 ± 0.03	0.229
Sphere(D)	-7.07 ± 0.95	-6.86 ± 1.00	0.316
Cylinder(D)	-0.98 ± 0.63	-0.68 ± 0.53	0.017
SE(D)	-7.56 ± 1.01	-7.20 ± 1.01	0.097
IOP (mmHg)	16.67 ± 2.65	15.84 ± 2.68	0.147
Axial length (mm)	26.48 ± 1.03	26.54 ± 0.94	0.756
CCT (µm)	545.69 ± 25.26	523.73 ± 30.61	0.000
OZ (mm)	6.10 ± 0.17	6.06 ± 0.14	0.200
AD (µm)	110.58 ± 12.43	105.93 ± 10.48	0.060
cET(µm)	53.20 ± 1.52	52.98 ± 1.44	0.479
mET (µm)	52.79 ± 1.53	52.79 ± 1.36	0.976
pET (µm)	51.84 ± 1.36	52.19 ± 1.22	0.210
KI front (D)	43.09 ± 1.07	42.93 ± 1.12	0.483
K2 front (D)	44.49 ± 1.37	44.14 ± 1.19	0.198
K1 Back (D)	-6.18 ± 0.20	-6.18 ± 0.21	0.951
K2 Back (D)	-6.61 ± 0.26	-6.59 ± 0.26	0.807
Regression time (months)	11.16 ± 3.18	11.83 ± 2.70	0.788

*UDVA* uncorrected distant visual acuity; *CDVA* corrected distance visual acuity; *SE* spherical equivalent; *logMAR* logarithm of the minimum angle of resolution; *D* diopters; *IOP* intraocular pressure; *CCT* central cornea thickness; *OZ* optical zone; *AD* ablation depth; *cET* central corneal epithelial thickness; *mET* paracentral epithelial thickness; *pET* midperipheral epithelial thickness; *K front* anterior corneal curvature; *K Back* posterior corneal curvature

**Fig. 1:**
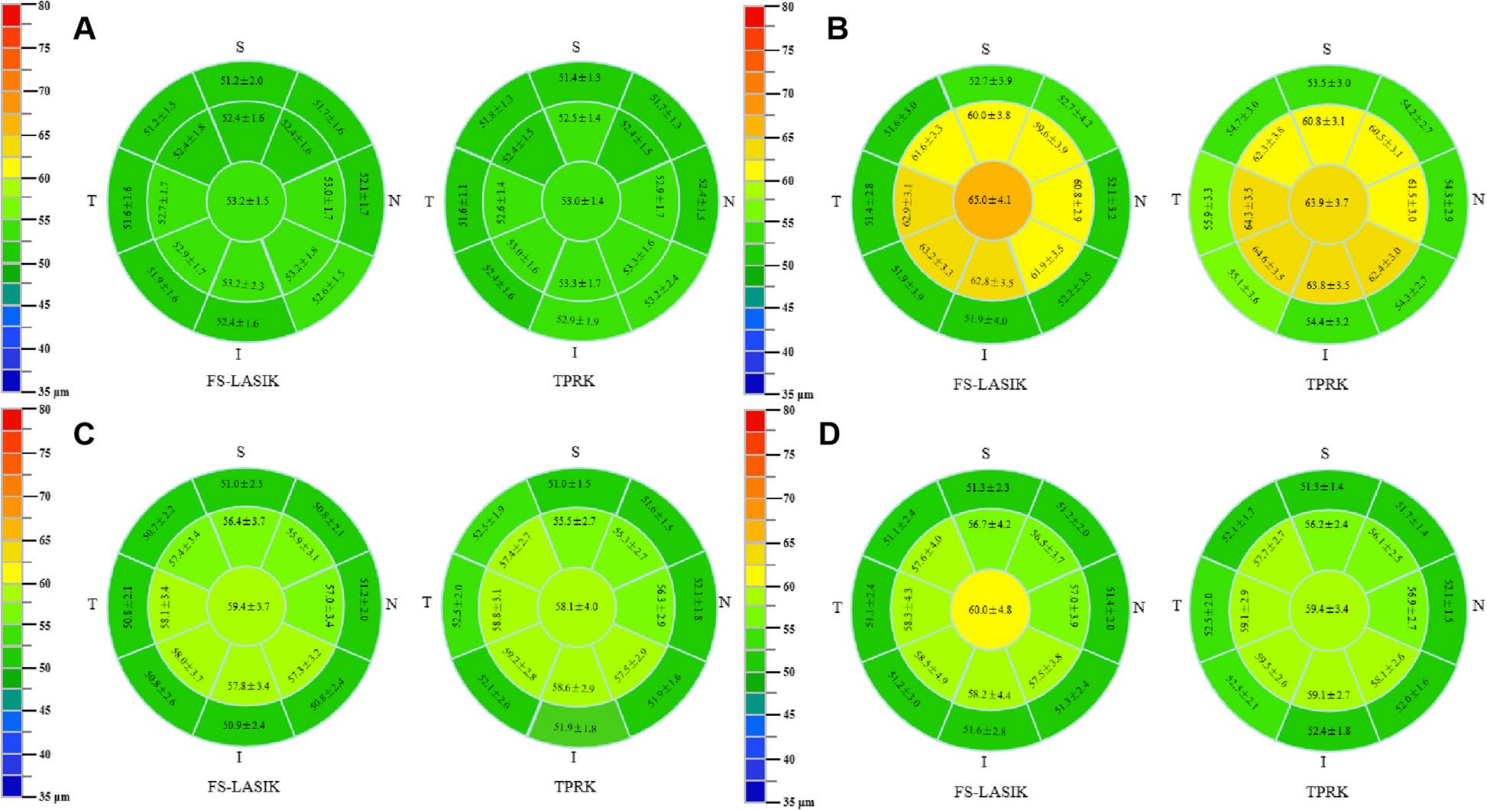
Schematic corneas displaying mean and standard deviation of corneal epithelial thickness in 17 sectors in the 7-mm diameter area in the FS-LASIK (*n* = 45 eyes) and TPRK (*n* = 44 eyes) groups before surgery (**A**), before drug treatment (**B**), after drug treatment (**C**), and after drug withdrawal (**D**)

When refractive regression occurred, the epithelial thickness of the two groups was 65.02 ± 4.12 µm (cET) and 61.63 ± 2.91 µm (mET) in FS-LASIK group and 63.89 ± 3.71 µm (cET), 62.52 ± 2.82 µm (mET), and 54.54 ± 2.23 µm (pET) in TPRK group, which was statistically different from that before surgery (*P* < 0.001; Fig. 1A-D, Table 2). However, the epithelial thickness in the midperipheral zone in FS-LASIK group (52.06 ± 2.97 µm) was not significantly different from that before surgery (*P* = 0.657). The thickness of the central corneal epithelium in both groups was more than 10 µm thicker than that before surgery when regression occurred. The spherical equivalent was - 0.92 ± 0.42 D in FS-LASIK group and - 0.64 ± 0.14 D in TPRK group (Table 2). The axial length was 26.46 ± 1.04 mm and 26.42 ± 0.96 mm in FS-LASIK and TPRK groups, respectively, which was not different from that before surgery (*P* > 0.05).

**Table 2:** Changes of ocular variables during regression in FS-LASIK and TPRK groups before treatment, after treatment and after drug withdrawal (Mean ± SD)

	FS-LASIK (*n* = 45 eyes)					TPRK (*n* = 44 eyes)				
	Initial	End	finish	*P*_*a*_	*P*_*b*_	Initial	End	finish	*P*_*a*_	*P*_*b*_
UDVA (logMAR)	0.15 ± 0.07	0.03 ± 0.06	0.03 ± 0.07	< 0.001	0.823	0.11 ± 0.04	-0.02 ± 0.04	-0.02 ± 0.04	< 0.001	0.710
CDVA (logMAR)	-0.02 ± 0.03	-0.02 ± 0.04	-0.02 ± 0.03	0.420	0.323	-0.03 ± 0.04	-0.03 ± 0.04	-0.03 ± 0.04	0.083	0.083
Sphere(D)	-0.81 ± 0.42	-0.11 ± 0.36	-0.14 ± 0.39	< 0.001	0.291	-0.53 ± 0.13	-0.02 ± 0.19	-0.05 ± 0.20	< 0.001	0.400
Cylinder(D)	-0.21 ± 0.17	-0.11 ± 0.16	-0.13 ± 0.26	0.001	0.679	-0.23 ± 0.18	-0.08 ± 0.13	-0.10 ± 0.13	< 0.001	0.372
SE(D)	-0.92 ± 0.42	-0.16 ± 0.37	-0.21 ± 0.46	< 0.001	0.251	-0.64 ± 0.14	-0.06 ± 0.19	-0.09 ± 0.21	< 0.001	0.220
IOP (mmHg)	12.00 ± 2.08	11.82 ± 2.02	11.89 ± 1.71	0.290	0.691	11.95 ± 1.41	11.95 ± 1.63	12.07 ± 1.44	1.000	0.342
Axial length (mm)	26.46 ± 1.02	26.46 ± 1.04	26.46 ± 1.04	0.069	0.719	26.42 ± 0.96	26.42 ± 0.95	26.42 ± 0.95	0.473	0.611
CCT (µm)	444.80 ± 28.40	437.31 ± 28.52	438.38 ± 27.62	< 0.001	0.130	414.98 ± 33.05	408.11 ± 34.81	408.93 ± 32.85	< 0.001	0.274
cET(µm)	65.02 ± 4.12	59.42 ± 3.70	60.02 ± 4.82	< 0.001	0.078	63.89 ± 3.71	58.05 ± 3.98	58.59 ± 3.20	< 0.001	0.095
mET (µm)	61.63 ± 2.91	57.24 ± 3.10	57.55 ± 3.97	< 0.001	0.253	62.52 ± 2.82	57.33 ± 2.53	57.67 ± 2.27	< 0.001	0.067
pET (µm)	52.06 ± 2.97	50.86 ± 1.85	51.26 ± 2.14	0.007	0.082	54.54 ± 2.23	51.93 ± 1.24	52.07 ± 1.11	< 0.001	0.286
KI front (D)	37.90 ± 1.10	37.10 ± 1.23	37.14 ± 1.18	< 0.001	0.200	37.37 ± 1.41	36.61 ± 1.52	36.64 ± 1.43	< 0.001	0.235
K2 front (D)	38.97 ± 1.15	38.07 ± 1.26	38.11 ± 1.27	< 0.001	0.140	38.36 ± 1.55	37.63 ± 1.68	37.65 ± 1.40	< 0.001	0.790
K1 Back (D)	-6.19 ± 0.21	-6.18 ± 0.20	-6.17 ± 0.20	0.561	0.110	-6.18 ± 0.21	-6.18 ± 0.21	-6.18 ± 0.22	0.352	0.323
K2 Back (D)	-6.57 ± 0.26	-6.59 ± 0.25	-6.58 ± 0.27	0.192	0.200	-6.58 ± 0.25	-6.57 ± 0.25	-6.57 ± 0.25	0.643	0.710

*SD* standard deviation; *UDVA* uncorrected distant visual acuity; *CDVA* corrected distance visual acuity; *SE* spherical equivalent; *logMAR* logarithm of the minimum angle of resolution; *D* diopters; *IOP* intraocular pressure; *CCT* central cornea thickness; *OZ* optical zone; *AD* ablation depth; *cET* central corneal epithelial thickness; *mET* paracentral epithelial thickness; *pET* midperipheral epithelial thickness; *K front* anterior corneal curvature; *K Back* posterior corneal curvature; *Initial* before drug treatment; *End* three months after drug treatment; *Finish* three months after drug withdrawal; _a _compared with before drug treatment; _b_ compared with three months after drug treatment

**Fig. 2:**
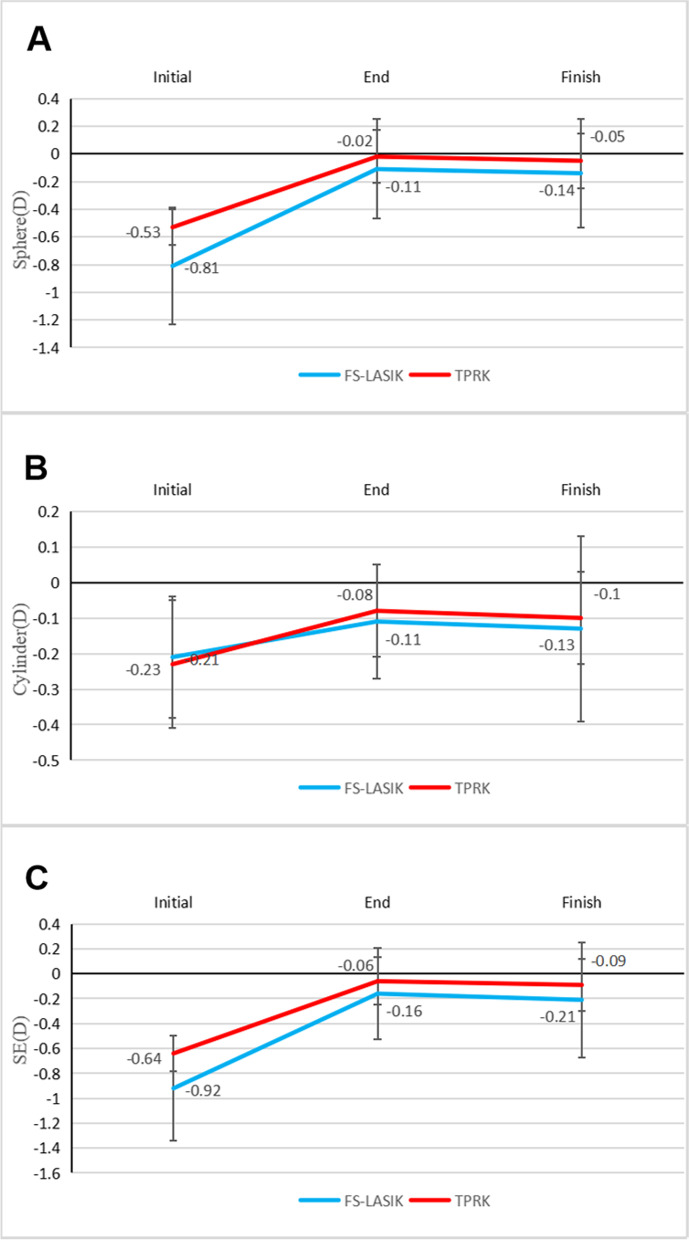
Changes of spherical (**A**), cylindrical (**B**), and spherical equivalent (**C**) in the FS-LASIK (*n* = 45 eyes) and TPRK (*n* = 44 eyes) groups before drug treatment, after drug treatment, and after drug withdrawal

**Fig. 3:**
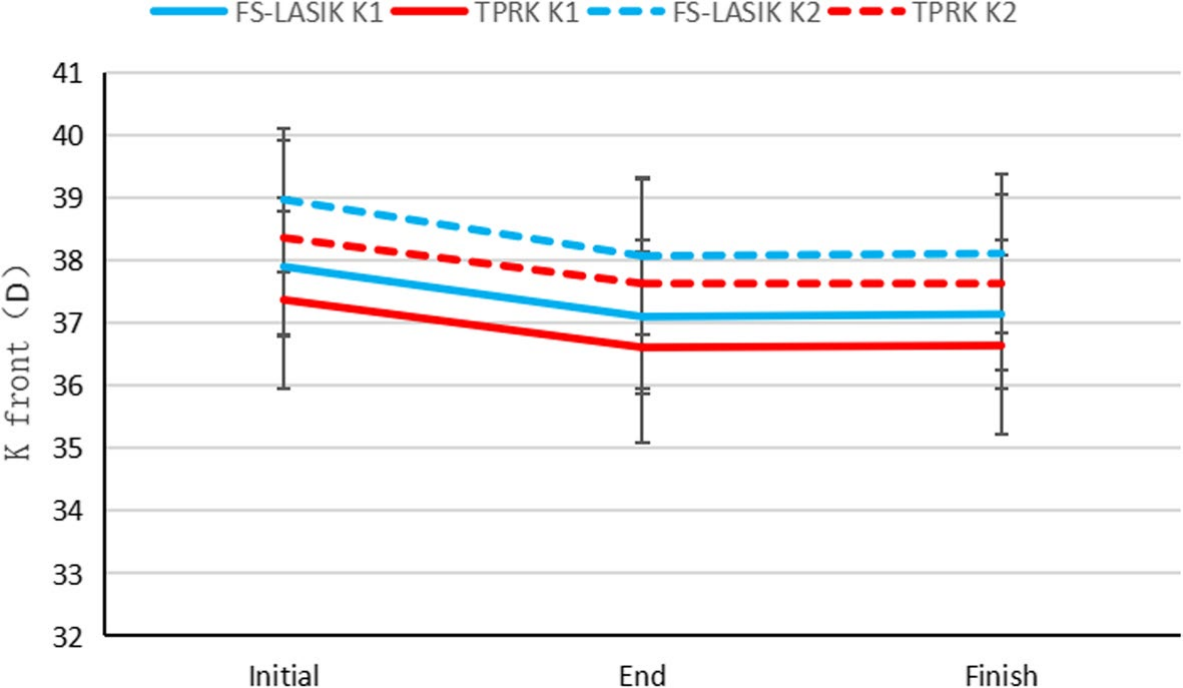
Changes of anterior corneal curvature (K1 and K2 front) in the FS-LASIK (*n* = 45 eyes) and TPRK (*n* = 44 eyes) groups before drug treatment, after drug treatment, and after drug withdrawal

**Fig. 4:**
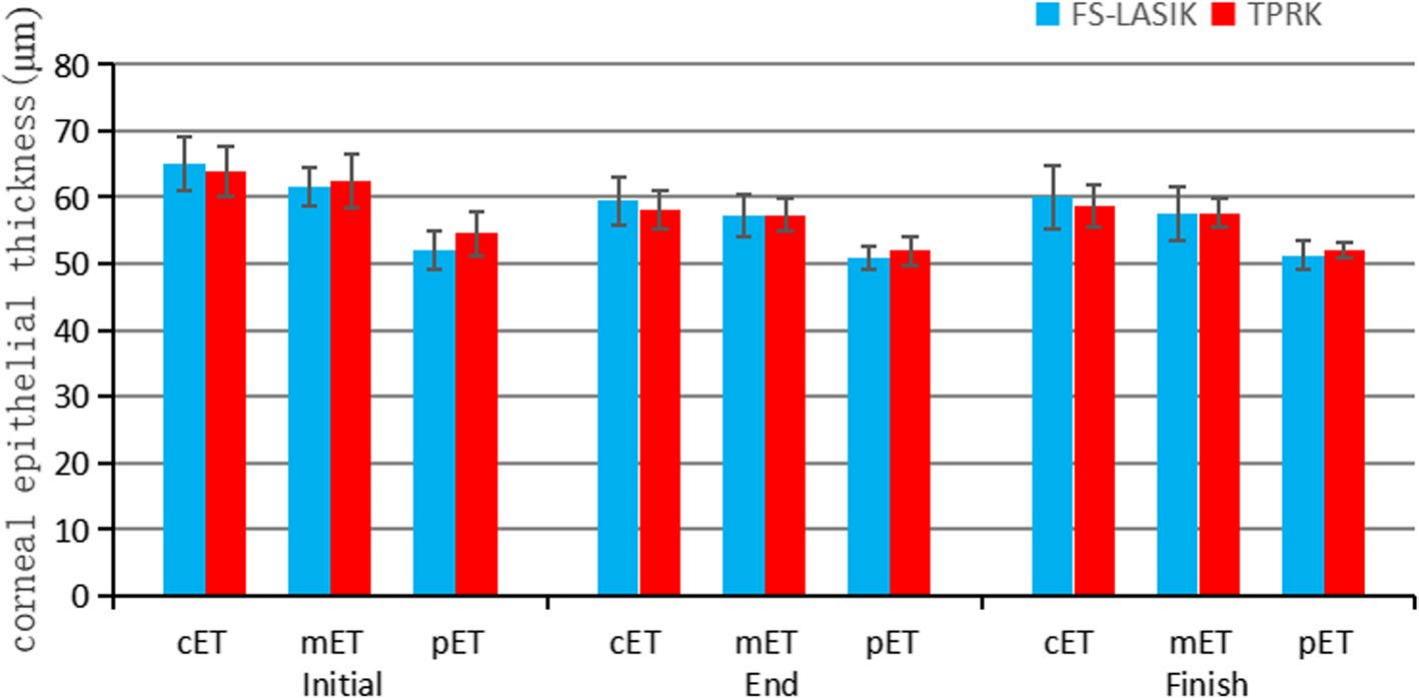
Changes of corneal epithelial thickness in the central (2 mm), paracentral (2 to 5 mm), and midperipheral (5 to 7 mm) zones separately before drug treatment, after drug treatment, and after drug withdrawal in the FS-LASIK (*n* = 45 eyes) and TPRK (*n* = 44 eyes) groups

After 3 months of drug treatment, there were significant differences in UDVA, spherical diopter, cylinderical diopter, spherical equivalent, central cornea thickness (CCT), front K1, front K2, and corneal epithelial thickness in three zones compared with the onset of regression (all *P* < 0.001; Table 2; Fig. 2A-C, Figs. 3 and 4). The thickness of the central corneal epithelium was reduced by approximately 6 µm compared to the onset of regression (Fig. 4). There were no significant differences in CDVA, intraocular pressure, axial length, and posterior corneal curvature. At 3 months after drug withdrawal, there were no significant differences in UDVA, CDVA, intraocular pressure, axial length, spherical diopter, cylinderical diopter, spherical equivalent, epithelial thickness, CCT, front K1, and front K2 compared with that after treatment (*P* > 0.05). There was no obvious thickening of corneal epithelium after 3 months of drug withdrawal (Table 2).

Significant differences were seen between the groups in terms of UDVA, sphere, spherical equivalent, CCT, pET, front K1, and front K2 (*P* < 0.05). Although there were no significant differences in cET and mET, there were significant differences in the change rate of epithelial thickness from the center to periphery. It was 12.96 ± 4.03 µm in FS-LASIK group and 9.35 ± 3.65 µm in TPRK group (*P* < 0.001). At 3 months after treatment and 3 months after treatment withdrawal, there were no differences between both groups in epithelial thickness, refractive error, and corneal curvature (*P* > 0.05).

## Discussion

It is a common phenomenon for the anterior surface areas flattened by laser ablation to be compensated by epithelial remodeling and thickening to maintain good optical quality of the cornea, after corneal reflective surgery [17, 18]. Variable patterns of epithelial thickening after surgery influence the postoperative refractive status differently [9]. When the epithelial thickening of the central regions exceeds the midperipheral regions, it will increase the optical power in the central regions, which is equivalent to adding a convex lens on the anterior corneal surface; therefore, refractive regression may occur. However, if the epithelial thickening of the midperipheral region exceeds the central regions, resulting in a negative meniscus-like epithelium, it is difficult for refractive regression to occur. Reinstein et al. found that the epithelium of the central regions thickened by 5 µm one month after LASIK for correcting myopia, refractive state drifting to myopia for 0.39 D [19]. The epithelial thickness increased by approximately 6 µm one year after surgery. Kanellopoulos showed that the epithelium thickened by 6 µm in the central regions one year after LASIK for correcting high myopia [20]. However, in the midperipheral regions, it was thickened by approximately 10 µm. Therefore, epithelial thickening does not necessarily cause refractive regression. In this study, epithelial thickening in central regions was more obvious than that in the midperipheral regions when refractive regression occurred.

The pattern of epithelial remodeling and thickening may be different between PRK and LASIK because the mechanism of corneal wound healing is different, and the probability and rate of refractive regression may also be different [21]. Earlier studies have reported that the duration and degree of epithelial thickening in PRK were different from that in LASIK [22]. However, in our previous studies, when correcting high myopia, the postoperative refractive status in FS-LASIK was more likely to drift to myopia than TPRK, which may be related to the more decreased biomechanical stability and more obvious epithelial thickening after FS-LASIK [23]. Kanellopoulos and Asimellis believed that the epithelial thickness was affected not only by the corneal curvature gradient but also by corneal biomechanics after surgery [24, 25]. The epithelial thickening is more obvious in patients with higher myopia and lower corneal thickness [22]. When FS-LASIK and PRK were combined with prophylactic cross-linking, corneal rigidity was strengthened, and the degree of epithelial thickening decreased; thus, the probability of refractive regression was reduced [26, 27].

This study prospectively investigated the postoperative refractive regression after TPRK and FS-LASIK for correcting high myopia. Refractive regression occurred approximately 1 year after surgery in most patients. The epithelial thickness was significantly higher than that before surgery when refractive regression occurred. In FS-LASIK group, the increase in epithelial thickness in the central region was more obvious than that in the midperipheral region, resulting in a greater degree of myopic regression. After 3 months of steroid treatment, the epithelial thickness was relatively lower and the refractive regression was reduced, which means that the occurrence of regression is highly correlated with epithelial thickening. This is similar to Ryu’s study [28]. However, in Ryu’s study, the preoperative epithelial thickness was lacking, and the direct relationship could not be obtained between the change in epithelial thickness and refractive status when regression occurred. Furthermore, due to the combined use of steroid and anti-glaucoma drugs, the corneal posterior surface shape might have been changed. Following this, the change of refractive state was affected. Our study also found that 3 months after drug treatment withdrawal, the refractive status remained relatively stable in most patients. However, regression in a few patients occurred again, accompanied by epithelial thickening and a decrease in visual acuity. Three patients with FS-LASIK needed enhancement surgery, and one patient undergoing TPRK needed surgical retreatment (data not reported). The reason for this difference is not clear. It was speculated that this difference is related to a higher decrease of biomechanical stability after FS-LASIK, suggesting the necessity for combining collagen cross-linking when correcting high myopia, especially in patients with high myopia and lower corneal thickness preoperatively [26, 27].

In addition to corneal epithelial hyperplasia and thickening, various studies consider that refractive regression is related to the protrusion of the posterior surface of the cornea [29–31]. Refractive regression can be treated with anti-glaucoma drugs by preventing the protrusion of the posterior surface [32, 33]. During clinical practice, topical steroidal agents and anti-glaucoma drugs were often used together to treat refractive regression, which could bring better therapeutic results because anti-glaucoma drugs could also reduce corneal epithelial thickness [34, 35]. However, in this study, to analyze the sole role of epithelial thickening during refractive regression, anti-glaucoma drugs were not used to prevent the protrusion of the posterior surface. Anti-glaucoma drugs were used in four eyes after the intraocular pressure increased by more than 5 mm Hg during steroid treatment. Meanwhile, we did not find that topical steroidal agents affected the posterior surface of the cornea. Therefore, the use of anti-glaucoma drugs in the four eyes would not affect the accuracy of the conclusions in this study.

There were limitations in our study. No control group not treated with drugs was designed. We could not confirm how long the refractive regression would last and whether it could subside by itself without intervention. In clinical practice, besides enhancement surgery, the main treatment of refractive regression was steroidal agents and anti-glaucoma drugs. Refractive regression will affect the visual quality of patients. For patients with refractive regression, if they were treated as a blank control and observed for 3 months, we could not obtain the consent of the patients, so we did not set up a control group. Our study is the before and after self-control study. The main purpose of the study is to use steroid treatment to change the thickness of the epithelium, observing the effect of the change of the epithelium thickness on refractive error, and then explaining the important role of the epithelium thickness in the refractive regression. It’s not about how effective the steroid treatment is, or whether it could subside by itself and how long it will last. Although there is no control group to explain the outcome of refractive regression, the changes of the epithelium before and after could explain the effect of epithelium thickness, which does not affect the elucidation of the role of epithelium in refractive regression. Compared with preoperative morphology, the corneal epithelium was indeed thickened with the central regions thickening more, which was different from that without regression in previous study. Epithelial thickening also exists in patients without regression. However, epithelial thickening in patients without refractive regression is concentrated in the inferior temporal region. Different from patients without regression, the epithelial thickness in the central area of cornea in patients with regression is significantly thicker. Furthermore, after steroidal treatment, the epithelium became thinner with the decreasing of curvature in corneal anterior surface, refractive error drifting to positive direction, regression subsiding and other ocular variables remained unchanged. It was enough to verify the purpose of this study, that was, the epithelial thickness played an important role in refractive regression, regardless of what factors leading to changes of epithelial thickness.

## Conclusion

In conclusion, this study demonstrated a close correlation between the corneal epithelium and refractive status. When refractive regression occurred, corneal epithelial thickness increased. Visual acuity and refractive status improved after steroid treatment, when epithelial thickness decreased.

## Data Availability

The datasets used and analysed during the current study are available from the corresponding author on reasonable request.

## Abbreviations

TPRK: Transepithelial photorefractive keratectomy
FS-LASIK: Femtosecond laser-assisted in situ keratomileusis
UDVA: Uncorrected distance visual acuity
CDVA: Corrected distance visual acuity
LASIK: Laser-assisted in situ keratomileusis
PRK: Photorefractive keratectomy
SMILE: Small-incision lenticule extraction
UDVA: Uncorrected distant visual acuity
CDVA: Corrected distance visual acuity
CCT: Central cornea thickness
cET: Central corneal epithelial thickness
mET: Paracentral epithelial thickness
pET: Midperipheral epithelial thickness
